# Flat-Band Localization in Electrical Circuits from One to Three Dimensions

**DOI:** 10.3390/ma19101981

**Published:** 2026-05-11

**Authors:** Kaixuan Shao, Feng Liu

**Affiliations:** School of Physics and Science Technology, Ningbo University, Ningbo 315000, China

**Keywords:** flat band, Lieb lattice, LC-resonant circuits, compact localized states

## Abstract

Flat bands exhibit vanishing group velocity and marked sensitivity to lattice geometry, making them a useful setting for studying localization driven by destructive interference. In this work, electrical-circuit simulations are employed to investigate flat-band systems in one, two, and three dimensions. A one-dimensional two-band circuit is first considered, and its flat-band response is characterized through node-to-ground impedance spectra and steady-state voltage distributions. The analysis is then extended to two- and three-dimensional Lieb lattice circuits characterized by sublattice imbalance. In the two-dimensional Lieb circuit, the flat band touches the dispersive bands at a Dirac point, so hybridization with dispersive modes affects the observed localization. Under periodic boundary conditions, wave vector quantization also produces responses that depend on whether the number of unit cells is even or odd. By contrast, in the three-dimensional Lieb circuit, the flat band is spectrally isolated from the dispersive bands, allowing stronger spatial confinement and clearer sublattice selectivity. The one-dimensional, two-dimensional, and three-dimensional models therefore represent three different situations: a singular flat band, a flat band that touches dispersive bands, and a spectrally isolated flat band. Comparing these cases shows how different degeneracy conditions shape impedance responses and localization patterns in electrical circuit systems. At the flat band frequency, the localized voltage response can also be used to generate spatial patterns in both two-dimensional and three-dimensional circuits, pointing to a possible route for spatial mode control of compact localized states in electrical systems.

## 1. Introduction

Flat bands are energy bands with vanishing dispersion that can arise in periodic lattices through destructive interference set by lattice geometry [1,2,3]. Because of their vanishing group velocity, flat band eigenstates are often compactly spatially localized, which makes these systems a useful setting for studying geometry-driven localization [4,5]. A central concept in this context is that of compact localized states (CLS), whose wavefunctions are strictly confined to a finite set of lattice sites due to destructive interference between hopping pathways [6,7,8,9]. When the flat band is spectrally isolated, CLS form a complete basis for the subspace of flat band, providing a simple and physically transparent description of localization. They therefore offer a useful framework for understanding the structure and origin of flat band eigenstates and have been widely studied in lattice systems of different dimensions [7,10,11,12]. However, the localization properties of flat bands can be strongly affected when the flat band is not spectrally isolated from dispersive bands. In particular, if a flat band touches a dispersive band, the degeneracy at the touching point may lead to singular or discontinuous behavior of the corresponding Bloch eigenfunctions. Flat bands with such band-touching singularities are commonly referred to as singular flat bands [13,14,15]. In these systems, the simple correspondence between flat bands and compact localized states can be modified, and a description based solely on ordinary compact localized states may become incomplete. As a result, the observed localization may deviate from the ideal compact-localization picture and can be modified by hybridization with dispersive-band modes. Therefore, distinguishing between spectrally isolated and singular flat bands is essential for understanding how band touching, eigenstate singularity, and mode hybridization influence flat-band localization.

Flat bands can arise either from engineered coupling patterns or from intrinsic lattice geometry. The Lieb lattice is a well-known example of the latter. Because it is a bipartite lattice with couplings only between different sublattices, it has chiral symmetry [16,17]. When the two sublattices contain different numbers of sites, zero energy modes appear, and their number is determined by the sublattice imbalance. This result was first established by Lieb in the Hubbard model and later generalized to noninteracting systems, where sublattice imbalance guarantees the existence of a zero-energy flat band [18]. This mechanism has recently been formulated in terms of chiral symmetry with nonequal sublattices, or CSNES, which offers a general way to understand flat band formation in such systems [19]. For this reason, the Lieb lattice has become a standard model for studying flat band physics and the associated localization properties.

Electrical circuits provide a flexible way to realize tight binding models and probe their spectral and spatial properties. In capacitor and inductor networks, Kirchhoff’s laws give rise to a grounded circuit Laplacian that acts as an effective Hamiltonian, whose eigenvalues and eigenvectors correspond to resonance frequencies and node voltage distributions [20,21,22,23,24]. This correspondence gives direct access to both band structures and eigenmodes. In addition, the impedance between nodes can be written in terms of the Laplacian eigenmodes, which makes impedance spectroscopy a useful probe of spectral features such as flat bands and their associated localized states [25,26,27,28,29]. Compared with conventional condensed matter systems, electrical circuits offer more flexible connectivity, tunable parameters, and convenient numerical modeling, making them well suited for studying flat band localization across multiple dimensions.

In this work, a one-dimensional two-band circuit model that hosts a flat band is first considered, and the study is then extended to Lieb lattice circuits in higher dimensions. In the one-dimensional circuit, the flat band is identified through node-to-ground impedance spectra and node voltage distributions, which reveal both its spectral signature and its localized character. Two-dimensional and three-dimensional Lieb lattice circuits with sublattice imbalance are then considered to examine how dimensionality and degeneracy affect the observed response. In the two-dimensional Lieb circuit, the flat band touches the dispersive bands at a Dirac point, so mode hybridization influences the observed localization. Under periodic boundary conditions, wave vector quantization also produces responses that depend on whether the number of unit cells is even or odd. By contrast, in the three-dimensional Lieb circuit, the flat band is separated from the dispersive bands in the spectrum, which makes the intrinsic localization of the flat band modes easier to resolve and leads to stronger spatial confinement and clearer sublattice selectivity. These comparisons show how different degeneracy conditions shape impedance responses and localization patterns in electrical circuit systems. It is also shown that the voltage distributions at the flat-band frequency can be used to generate spatial patterns in both two-dimensional and three-dimensional circuits.

## 2. Methods

This study is based on LTspice XVII AC simulations and MATLAB R2025a calculations. LTspice is used to build the circuit networks and simulate their AC responses, including node-to-ground impedance spectra and steady-state node voltage distributions under harmonic driving. MATLAB is used to construct the grounded circuit Laplacian and solve the corresponding eigenvalue problem, which yields the eigenfrequencies, band structures, and eigenmodes of finite systems. For periodic systems, the circuit Laplacian is also constructed in momentum space and diagonalized along high symmetry paths in the Brillouin zone to obtain the band dispersions. Electrical realizations of one-, two-, and three-dimensional lattice systems are considered. The one-dimensional system is modeled as a ring circuit containing 16 unit cells. The two-dimensional Lieb lattice is implemented as a periodic circuit network; a 12 × 12 lattice is used for the main voltage-distribution calculations, while additional odd- and even-sized lattices are considered when analyzing parity effects under periodic boundary conditions. The three-dimensional Lieb lattice is modeled as a periodic structure consisting of 6 × 6 × 7 unit cells. These system sizes are chosen as a compromise between numerical tractability and adequate resolution of the spectral and spatial characteristics of the flat-band modes. The circuit connectivity follows the topology of the corresponding lattice model, and each unit cell contains inequivalent nodes representing different sublattice degrees of freedom.

In the LTspice AC simulations, the node-to-ground impedance is obtained from the simulated voltage and current at a selected node under harmonic excitation. In practice, the excitation is typically implemented by a current source connected to the target node, and a small series resistor may be introduced when needed. The simulations indicate that the resistance value of this series resistor has little influence on the measured impedance spectra within the parameter range considered here. Unless otherwise specified, the AC amplitude of the current source is set to 1, so that the simulated voltage response can be directly used to evaluate the impedance. The frequency range and frequency step can be adjusted according to the specific circuit parameters and the desired spectral resolution. To probe the spatial structure of the modes, the circuit is driven at a specified frequency and the simulated voltage amplitudes |V| at all nodes are extracted to generate the voltage distributions. For visualization and comparison, the voltage distributions are normalized by the maximum node voltage in each configuration, such that the displayed values lie in the range [0, 1]. Unless otherwise stated, all voltage maps shown in this work correspond to these normalized amplitudes. By comparing the simulated impedance spectra and normalized voltage distributions at the flat-band frequency and at selected off-resonant frequencies, the flat-band response and the associated localization behavior can be identified. Ideal capacitors and inductors are used throughout the simulations. The component values of the capacitors and inductors were chosen to place the flat-band resonances within a suitable frequency range. In the circuit Laplacian, capacitive couplings determine the effective hopping strengths, whereas grounded inductive and capacitive elements control the onsite resonant response. Thus, the relative component ratios mainly determine the band structure and localization behavior. Realistic nonidealities, including equivalent series resistance, contact resistance, and component tolerances, may reduce the quality factor of the resonant response, broaden the impedance peaks, and cause small shifts in the resonance frequencies. They may also introduce weak spatial inhomogeneity in the voltage distributions. Nevertheless, when these effects remain sufficiently small, the qualitative flat-band localization pattern is expected to be preserved because it is primarily governed by the destructive-interference condition of the lattice geometry. For the three-dimensional model, active modules such as operational amplifiers and voltage-controlled current sources are introduced in LTspice when required to realize the designed coupling terms.

## 3. Results

### 3.1. One-Dimensional Chain with Two Energy Bands

A one-dimensional lattice model that supports a flat band is first constructed. Consider a general two-band Hamiltonian of the form

(1) $$H_{k}=\left(\begin{array}{cc} A & B\\ B^{*} & D \end{array}\right),$$

where A and D are real-valued functions of the wave vector k, while B is generally complex. If the system supports a flat band at E = 0, the characteristic equation requires

(2) $$AD={\left|B\right|}^{2}.$$

Under this condition, one has $\left|B\right|=\sqrt{AD}$, and the eigenvalues can be written as

(3) $$E_{FB}=0,E_{dis}=A+D.$$

The Hamiltonian can then be parametrized as

(4) $$H_{k}=\left(\begin{array}{cc} A & -te^{i\theta \left(k\right)}\\ -te^{-i\theta \left(k\right)} & D \end{array}\right),$$

where $t=\sqrt{AD}$ and e^iθ(k)^ is an arbitrary phase factor. The corresponding eigenstates can be written as

(5) $$\psi_{FB}=\left(\begin{array}{c} te^{i\theta }\\ A \end{array}\right),\;\psi_{dis}=\left(\begin{array}{c} te^{i\theta }\\ -D \end{array}\right).$$

To realize specific hopping configurations between the two sublattices, the off-diagonal term may be expanded as a Fourier series,

(6) $$te^{i\theta \left(k\right)}=\sum_{n=0}^{\infty }\lambda_{n}e^{-ikn},$$

As a concrete example of a one-dimensional singular flat-band model, the following Hamiltonian is chosen:

(7) $$B=-t\left(1+e^{-ik}\right),$$

This choice corresponds to the minimal two-term Fourier expansion and ensures that B vanishes at *k* = π, which is essential for generating singular behavior in the flat-band eigenstates. Substituting Equation (7) into the flat-band condition gives

(8) $$AD={\left|B\right|}^{2}=t^{2}\left(2+2\;\cos k\right)=4{\;\cos}^{2}\left(k/2\right),$$

A symmetric and minimal choice satisfying this constraint is A=D=±2cos(k/2). However, the appearance of cos(k/2) indicates that this representation is not periodic under *k* → *k* + 2π but instead has an effective periodicity of 4π. This implies that the underlying real-space description corresponds to an enlarged unit cell. To restore the standard Bloch periodicity, the unit cell is enlarged by a factor of two. Under this redefinition, the Hamiltonian can be rewritten as

(9) $$H_{k}=-t\left(\begin{array}{cc} 2\cos k & 1+e^{-2ik}\\ 1+e^{2ik} & 2\cos k \end{array}\right),$$

**Figure 1:**
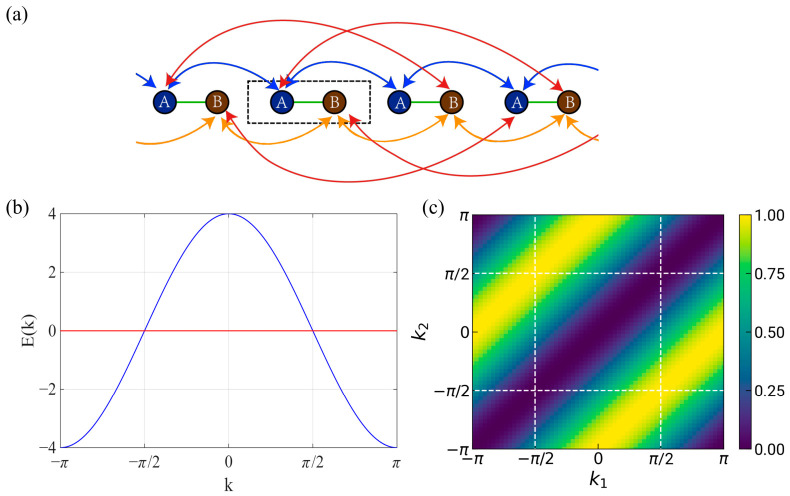
(**a**) Schematic illustration of the singular 1D flat-band model described by Equation (9), where the arrows denote hopping of amplitude −t and the dashed rectangle marks the unit cell. (**b**) Band structure of the singular 1D flat-band model. (**c**) Quantum distance of the flat band, with the singular points indicated by the white dashed line. The quantum distance reaches its maximum value of 1 for *θ*1 − *θ*2 = π around π/2 with $k_{1}=\frac{\pi }{2}e^{i\theta_{1}}$ and $k_{2}=\frac{\pi }{2}e^{i\theta_{2}}$.

In this form, the Hamiltonian is explicitly periodic under *k* → *k* + 2π, yielding a consistent lattice description of a one-dimensional flat-band system. The schematic corresponding to the lattice model of Equation (9) is shown in Figure 1a.

By solving the eigenvalue problem of *H_k_*, one obtains a band structure composed of a flat band and a dispersive band, with E_FB_ = 0, E_dis_ = 4cosk, as illustrated in Figure 1b. The corresponding eigenvector of the flat band is given by

(10) $$\psi_{FB}=c_{k}\left(\begin{array}{c} -1-e^{-2ik}\\ 2\cos k \end{array}\right),$$

where the normalization factor is

(11) $$c_{k}=\frac{1}{2\sqrt{2}|\cos k|}.$$

At the degeneracy point k = π/2, one finds that $\psi_{FB}=0.$ The quantum distance d_k_ is defined as

(12) $$d^{2}\left(k_{1},k_{2}\right)=1-{\left|\left\langle \psi_{FB}\left(k_{1}\right),\psi_{FB}\left(k_{2}\right)\right\rangle \right|}^{2},$$

Substitution of the flat-band eigenstates into the Hilbert–Schmidt distance d^2^(k_1_, k_2_) gives:

(13) $$\left\langle \psi_{FB}\left(k_{1}\right),\psi_{FB}\left(k_{2}\right)\right\rangle =\frac{\left(1+e^{2ik_{1}}\right)\left(1+e^{-2ik_{2}}\right)+4\cos k_{1}\;{cos\;k}_{2}}{8\left|\cos k_{1}\right|\left|\cos k_{2}\right|}.$$

By plotting the absolute value of Equation (13) as a function of *k*_1_ and *k*_2_, the corresponding results can be obtained. As shown in Figure 1c, the quantum distance reaches its maximum value of 1 near |*k*| = π/2, indicating that the one-dimensional flat band is singular [30].

**Figure 2:**
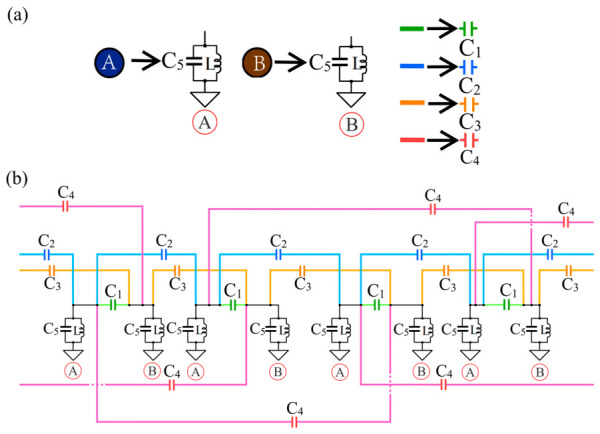
(**a**) Correspondence between the atomic structure of the one-dimensional chain and its circuit realization in Figure 1a. The green lines denote the hopping between A and B atoms within the same unit cell, the blue lines denote the nearest-neighbor intercell hopping between A atoms, the orange lines denote the nearest-neighbor intercell hopping between B atoms, and the red lines denote the next-nearest-neighbor intercell hopping between A and B atoms. (**b**) Schematic diagram of the one-dimensional chain circuit.

Based on this structure, a corresponding circuit model is constructed, and the correspondence between the lattice sites in the atomic structure and the circuit elements is illustrated in Figure 2a Meanwhile, a one-dimensional two-band model with periodic boundary conditions is implemented in the circuit. Specifically, the one-dimensional chain containing 16 unit cells is connected end to end to form a closed ring, thereby realizing periodic boundary conditions in the circuit.

Based on Kirchhoff’s laws, the Laplacian matrix corresponding to a single unit cell can be derived, leading to the circuit equation *I*(*ω*) = *J*(*ω*)*V*(*ω*). Since the circuit is a passive network, *I*(*ω*) = 0 can be taken in the absence of external AC current injection, thereby transforming the problem into an eigenvalue equation. After further derivation, one obtains

(14) $$J\left(\begin{array}{c} V_{1}\\ V_{2} \end{array}\right)=\frac{1}{\omega^{2}L}\left(\begin{array}{c} V_{1}\\ V_{2} \end{array}\right),$$

(15) $$J=\left(\begin{array}{cc} 2CL\cos k\;+\;8CL\; & \;-CL\left(1\;+\;e^{-2ik}\right)\;\\ -CL\left(1\;+\;e^{2ik}\right)\; & \;2CL\cos k\;+\;8CL \end{array}\right),$$

**Figure 3:**
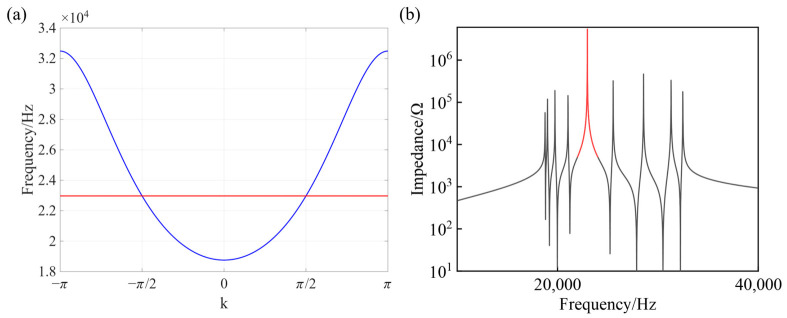
(**a**) Circuit frequency as a function of wave vector k. (**b**) Node impedance as a function of frequency, where the impedance spectra exhibit identical peak values at the two nodes within each unit cell; the red region corresponds to the impedance peaks associated with the frequency band in the vicinity of the flat-band frequency.

After solving Equation (15), the eigenvalues can be obtained as *λ* = *1*/*ω*^2^*L*, which vary as a function of the wave vector *k*∈(−π, π), where *C* = 1 nF and *L* = 6 mH. Using the relation $f=\sqrt{L}/(2\pi \sqrt{\lambda })$, the corresponding frequency dispersion as a function of *k* can be calculated, as shown in Figure 3a. The results clearly indicate the presence of a flat band in the system, with a corresponding frequency of approximately 22,972 Hz.

To characterize the spectral properties of the circuit system, a frequency-resolved analysis is first performed by calculating the node-to-ground impedance spectrum. Specifically, a single node is selected, and its impedance to ground is calculated as a function of frequency, yielding the impedance spectrum shown in Figure 3b. The results reveal a pronounced resonance peak at a specific frequency, which coincides with the flat-band frequency of the system, indicating that the flat-band mode manifests as a distinct feature in the impedance response. To further probe the spatial characteristics of the eigenmodes, the node voltage distribution is introduced as a complementary diagnostic. Owing to the correspondence between circuit theory and the tight-binding model, the node voltages can be interpreted as the amplitudes of the eigenstates of an effective Hamiltonian on the lattice sites. Therefore, by exciting the circuit at a given frequency and measuring the voltages at all nodes, one can directly visualize the spatial structure of the corresponding eigenmode.

**Figure 4:**
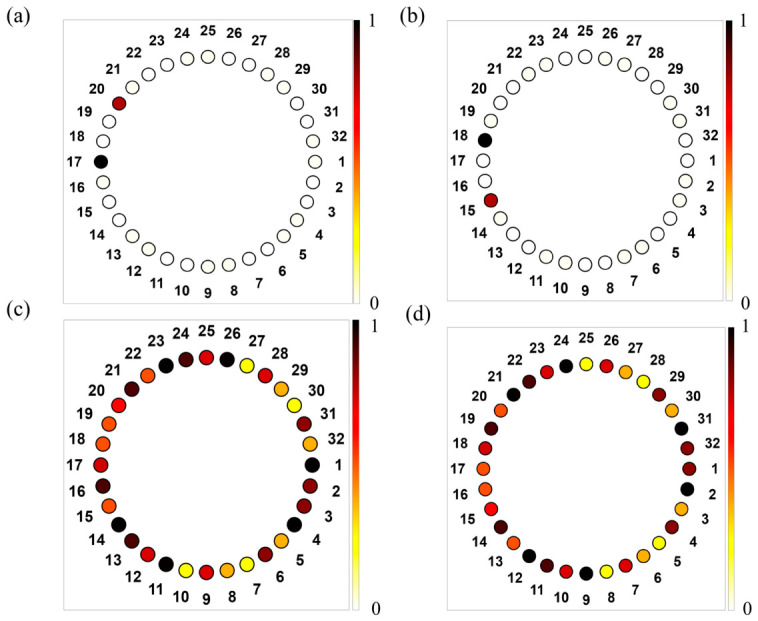
(**a**,**b**) show the normalized voltage amplitude distributions under excitation at node 17 and node 18 at the flat-band frequency of 22,972 Hz, respectively. (**c**,**d**) show the corresponding normalized voltage distributions at the same nodes under excitation at 30,000 Hz.

In the simulations, nodes 17 and 18 are chosen as excitation points, corresponding to the A and B sublattices of the one-dimensional structure, respectively. When the excitation frequency is set to the flat-band frequency, an AC source is applied to each of these nodes in turn, and the resulting voltage distributions are shown in Figure 4a,b. The results demonstrate that the voltage is predominantly localized around the excitation site, with only weak responses appearing on neighboring sites of the opposite sublattice in adjacent unit cells, while the amplitudes on all other nodes are nearly negligible. This behavior clearly indicates strong spatial localization. These observations confirm that, at the flat-band frequency, the system supports highly localized eigenmodes whose amplitudes are confined to a finite number of lattice sites. For comparison, when the excitation frequency is detuned from the flat-band frequency to 30,000 Hz while keeping the same excitation protocol, the voltage distributions, as shown in Figure 4c,d, extend over the entire circuit rather than remaining localized. This contrast further confirms that the observed localization originates from the intrinsic flat-band eigenstates, rather than from the excitation scheme or boundary effects. To quantitatively evaluate the degree of spatial localization of the circuit modes, the inverse participation ratio (IPR) is calculated from the simulated node-voltage amplitudes. For the voltage distribution *V_i_* over all circuit nodes, the IPR is defined as follows:

(16) $$\mathrm{IPR}=\frac{\sum_{i}{\left|V_{i}\right|}^{4}}{{\left(\sum_{i}{\left|V_{i}\right|}^{2}\right)}^{2}}$$

where V_i_ denotes the voltage amplitude at the (i)-th node. A larger IPR indicates that the voltage response is concentrated on fewer nodes, corresponding to stronger localization, whereas a smaller IPR indicates a more extended voltage distribution. The calculated IPR values are 0.44 for excitation at 22,972 Hz and 0.04 for excitation at 30,000 Hz. These results further provide quantitative evidence that the voltage distribution is strongly localized at the flat-band frequency.

### 3.2. Two-Dimensional Lieb Lattice

**Figure 5:**
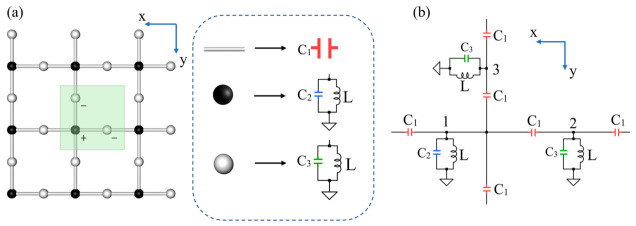
Circuit design of the two-dimensional Lieb lattice. (**a**) Schematic of a two-dimensional Lieb lattice composed of 3 × 3 unit cells, Inside the green area is a single unit cell. (**b**) Circuit implementation of a single unit cell. The onsite potentials of the two sublattices are realized by grounded LC resonant circuits, while the hopping between neighboring lattice sites is implemented by the coupling capacitor *C*_1_. The unit cell contains three nodes labeled 1, 2, and 3. The circuit parameters are $C_{1}=5\;uF,{\;C}_{2}=1\;uF,{\;C}_{3}=11\;uF,\;L=1\;mH$.

To investigate structures with chiral symmetry and non-equal sublattices (CSNES), the classical two-dimensional Lieb lattice is adopted as the model system. The two-dimensional Lieb lattice can be regarded as a square lattice, in which an additional site is inserted between two neighboring lattice sites, as shown in Figure 5a [31,32,33].

Each unit cell contains one corner site and two edge-centered sites, forming a bipartite network with an unequal number of sublattice sites. To realize the two-dimensional Lieb lattice in an electrical circuit, one type of capacitor and two types of grounded LC resonant circuits are employed. The capacitors are used to simulate the hopping coupling between lattice sites, while the two grounded LC resonant circuits are used to simulate two different onsite potentials. The nodes are connected by identical capacitors along the x and y directions, thereby reproducing the coupling structure of the two-dimensional Lieb lattice, the unit-cell circuit of the two-dimensional Lieb lattice is shown in Figure 5b. After introducing the two-dimensional Lieb lattice, the mathematical method used to analyze its spectral properties is presented. Kirchhoff’s law is applied to the circuit unit cell, where the node voltages V and currents I are defined. To calculate the band structure, an infinite two-dimensional periodic lattice is considered, where *q_x_* and *q_y_* denote the Bloch wave phases propagating along the x and y directions, respectively. In such periodic circuits the voltage vector *V* and the current vector *I* are related through the grounded circuit Laplacian matrix Equation (17).

(17) $$\left(\begin{array}{c} I_{1}\\ I_{2}\\ I_{3} \end{array}\right)=J\left(\begin{array}{c} V_{1}\\ V_{2}\\ V_{3} \end{array}\right).$$

The admittances of the capacitor and inductor are given by Equation (18)

(18) $$y_{C}\;=\;i\omega C\;,\;y_{L}\;=\;\frac{1}{i\omega L},$$

where the matrix Y is expressed as Equation (19)

(19) $$Y=\left(\begin{array}{ccc} C_{2}\;+\;4C_{1}\; & \;-C_{1}\;\left(\;e^{-i\;q_{x}}\;+\;1\;\right)\; & \;{-C}_{1}\;\left(\;e^{-i\;q_{y}}\;+\;1\;\right)\;\\ {-C}_{1}\;\left(\;e^{i\;q_{x}}\;+\;1\;\right)\; & \;C_{3}\;+\;2C_{1}\; & \;0\;\\ {-C}_{1}\;\left(\;e^{i\;q_{y}}\;+\;1\;\right)\; & \;0\; & \;C_{3}\;+\;2C_{1} \end{array}\right),$$

**Figure 6:**
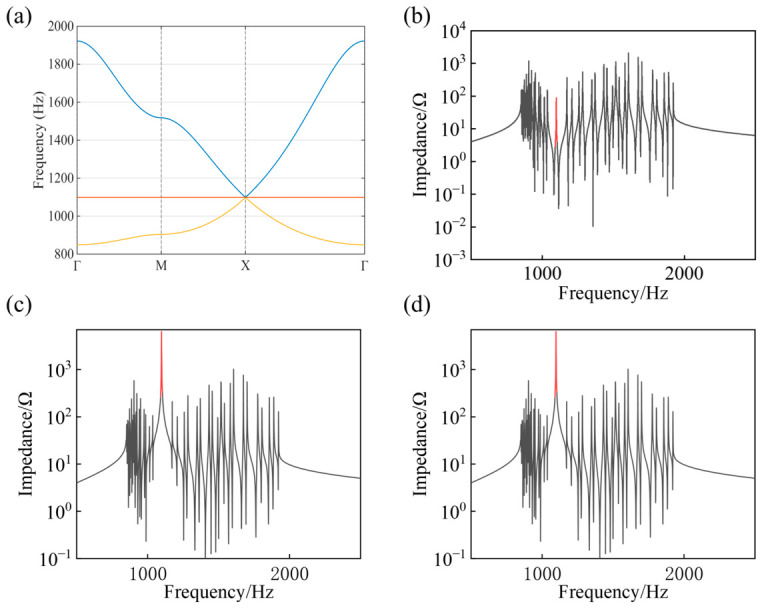
Theoretical frequency spectrum and node impedance responses of the two-dimensional Lieb lattice circuit. (**a**) Eigenfrequency band structure calculated from the grounded circuit Laplacian matrix of a single unit cell derived from the periodic circuit shown in Figure 5b. The band structure is obtained along the high-symmetry path Γ(0,0) → X(π,0) → M(π,π) → Γ(0,0) in the Brillouin zone with circuit parameters $C_{1}=5\;uF,{\;C}_{2}=1\;uF,C_{3}=11\;uF,L=1\;mH$. (**b**–**d**) Node-to-ground impedance spectra calculated by applying an AC excitation signal to nodes 1, 2, and 3 of the same circuit unit cell, respectively, using a two-point impedance simulation approach. The red region indicates a narrow frequency band around the flat-band frequency.

The diagonal elements have clear physical origins. Node 1 is connected to four neighboring nodes through capacitors *C*_1_ and to the grounded capacitor *C*_2_, which results in the term *C*_2_ + 4*C*_1_. In contrast, nodes 2 and 3 are connected to only two neighboring nodes and to grounded capacitors *C*_3_, giving the terms *C*_3_ + 2*C*_1_. The phase factors $e^{\pm iq_{x}}$ and $e^{\pm iq_{y}}$ originate from the Bloch boundary conditions imposed on the periodic lattice. By substituting the circuit parameters $C_{1}=5\;uF,{\;C}_{2}=1\;uF,{\;C}_{3}=11\;uF,L=1\;mH$ into the matrix *Y*, the eigenvalues of the matrix are calculated. From the obtained eigenvalues, the corresponding eigenfrequencies ω are determined. The circuit frequencies are then obtained through f = ω/ 2π. The resulting frequency spectrum along the high-symmetry path Γ(0,0) → X(π,0) → M(π,π) → Γ(0,0) is shown in Figure 6a. Three bands appear in the spectrum, where the middle band corresponds to a flat band with a frequency of 1098 Hz. The flat band touches the dispersive bands at the X point of the Brillouin zone, forming a Dirac point that is characteristic of the Lieb lattice band structure [17].

To perform numerical simulations of the circuit response, an AC voltage source connected in series with a resistor is applied to individual nodes and the node-to-ground impedance spectra are calculated. The impedance responses calculated at nodes 1, 2 and 3 are shown in Figure 6b–d. According to the CSNES mechanism, different sublattices are expected to exhibit different responses. As shown in Figure 6c,d, pronounced impedance peaks appear at the flat-band frequency for nodes 2 and 3, indicating strong resonant responses associated with the flat-band modes.

However, a smaller impedance peak can also be observed at node 1 in Figure 6b. Since the band structure of the two-dimensional Lieb lattice contains a Dirac point, where the flat band touches the dispersive bands, the eigenmodes near the band-crossing point may involve hybridization between flat-band modes and dispersive Dirac modes. As a result, a finite response can appear at node 1 even though the ideal flat-band mode is mainly localized on nodes 2 and 3.

**Figure 7:**
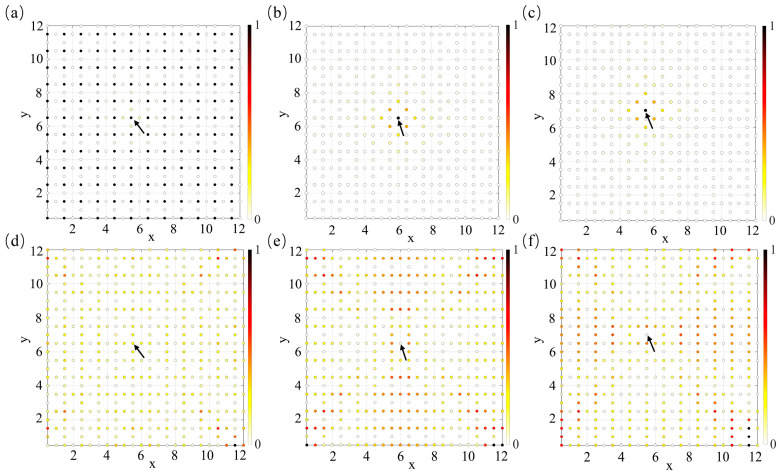
Spatial distributions of normalized node voltage amplitudes in a 12 × 12 unit-cell periodic two-dimensional Lieb lattice circuit under fixed-frequency excitation, the black arrows indicate the excitation nodes. (**a**,**d**) Voltage distributions when the excitation is applied to node 1 of a unit cell at lattice coordinate (5.5, 6.5). Panel (**a**) corresponds to the flat-band frequency 1098 Hz, while panel (**d**) corresponds to an off-flat-band frequency 1500 Hz. (**b**,**e**) Voltage distributions when the excitation is applied to node 2 at (6, 6.5). Panel (**b**) corresponds to 1098 Hz and panel (**e**) to 1500 Hz. (**c**,**f**) Voltage distributions when the excitation is applied to node 3 at (5.5, 7). Panel (**c**) corresponds to 1098 Hz and panel (**f**) to 1500 Hz.

To further examine the spatial characteristics of the circuit modes, the voltage distributions under node excitation are calculated as an independent verification of the localization behavior. According to the correspondence between the circuit Laplacian equation and the lattice Hamiltonian, the node voltages correspond to the eigenvectors of the circuit modes. The amplitude of the eigenvector reflects the distribution of the corresponding mode over the circuit nodes. Therefore, the spatial distribution of the node voltage amplitudes under excitation can be used to characterize the spatial properties of the circuit modes and to determine whether localization occurs. Based on this principle, the voltage distributions obtained under excitation at different nodes are shown in Figure 7. When nodes 2 and 3 are excited at the flat-band frequency, the voltage amplitudes are mainly concentrated around the excitation position, indicating a localized state. In contrast, as shown in Figure 7a, excitation at node 1 does not produce a localized voltage distribution at the flat-band frequency. Furthermore, when the excitation frequency deviates from the flat-band frequency (1500 Hz), the voltage distributions shown in Figure 7d–f do not exhibit localization for excitations at any of the three nodes. These results indicate that, in the Lieb lattice circuit, localization occurs only when the excitation is applied at the flat-band frequency and injected at nodes 2 and 3, as shown in Figure 7b,c. Moreover, the calculated IPR values further reveal the sublattice-dependent localization behavior. At the flat-band frequency of 1098 Hz, the IPR obtained for excitation at node 1 is only 0.007, whereas those obtained for excitation at nodes 2 and 3 are both 0.28, indicating that excitations on nodes 2 and 3 generate much more localized voltage responses. In contrast, at the off-flat-band frequency of 1500 Hz, the IPR values are 0.009 for excitation at node 1 and 0.008 for excitations at nodes 2 and 3, suggesting that the voltage distributions are more extended away from the flat-band resonance.

For the circuit structure under consideration, the single-unit-cell circuit with periodic boundary conditions is systematically extended to a 16 × 16 unit-cell lattice, and its spectral response is analyzed in detail. The results demonstrate that, under periodic boundary conditions, systems composed of an odd number of unit cells and those composed of an even number of unit cells exhibit qualitatively distinct behaviors in the characterization calculated at node 1. To account for this phenomenon, it is crucial to recognize that, under periodic boundary conditions, the wave vectors k in the circuit become discretized due to quantization, with their allowed values satisfying

(20) $$k_{x}=\frac{2\pi m}{N_{x}a_{x}},k_{y}=\frac{2\pi n}{N_{y}a_{y}},$$

(21) $$m=0,1,\ldots ,N_{x}-1,n=0,1,\ldots ,N_{y}-1.$$

Here, N_X_ and N_y_ denote the numbers of unit cells along the (x)- and (y)-directions, respectively. According to the above relation, only when both N_X_ and N_y_ are even can the set of allowed discrete (*k*)-points include the high-symmetry point M(π,π). From this, it can be further concluded that the flat band does not exhibit any state weight on node 1; moreover, in systems with an even number of unit cells, the peak observed at the flat-band frequency at node 1 does not originate from the flat band itself but rather arises from the inclusion of the Dirac point in the discrete (*k*)-space, corresponding to the projection of the Dirac-point states onto node 1. A more quantitative discussion of the influence of the band-touching point is provided in Supplementary Material S1.

**Figure 8:**
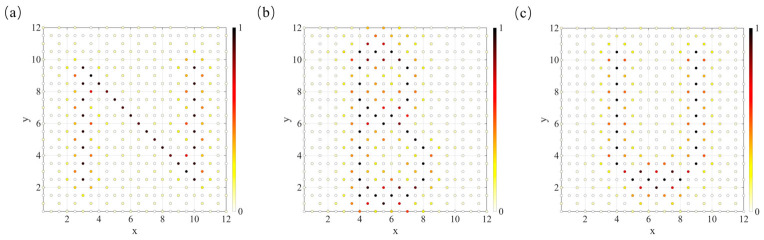
(**a**) Excitation result for the letter “N”. (**b**) Excitation result for the letter “B”. (**c**) Excitation result for the letter “U”.

The voltage localization associated with compact localized states shows that, when in-phase excitations are applied at nodes 2 and 3, the system exhibits a pronounced compact-localization character; the voltage is predominantly confined to the vicinity of the excitation sites, with only weak responses in the neighboring region, while the voltages at all other nodes remain nearly vanishing. This strong spatial confinement further suggests the potential applicability of such localized responses. In particular, by appropriately tailoring the positions and configurations of the in-phase excitation voltages, the post-excitation voltage profile can be controllably manipulated so as to generate prescribed spatial patterns. In the present work, specific excitation schemes are designed on the basis of this voltage-localization effect, such that the resulting voltage distributions reproduce the letters N, B, and U, as shown in Figure 8.

### 3.3. Three-Dimensional Lieb Lattice Circuit

**Figure 9:**
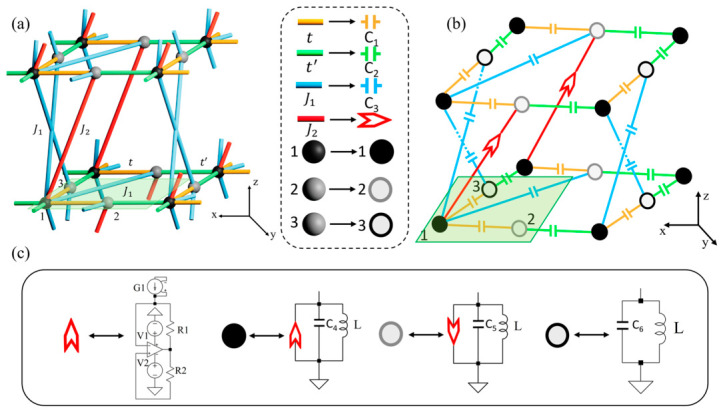
Circuit design of the three-dimensional Lieb lattice. (**a**) Schematic of the three-dimensional Lieb lattice formed by stacking two-dimensional Lieb lattices along the z direction. Each unit cell contains two sublattices with hopping only between different sublattices, preserving chiral symmetry. The in-plane couplings are t, $t^{\prime }$ and $J_{1}$, while interlayer couplings are $J_{1}$ and $J_{2}$. The parameters are t=0.5, $t^{\prime }=1$, $J_{1}=1$, and $J_{2}\;$= *i* [19]. (**b**,**c**) Circuit representation and implementation of a unit cell. Grounded LC resonators realize the onsite potentials, capacitors *C*_1_, *C*_2_ and *C*_3_ implement the couplings t, $t^{\prime }$ and $J_{1}$, and the complex coupling $J_{2}$ is realized by an operational-amplifier module with a voltage-controlled current source (VCCS). The circuit parameters are $C_{1}=5\;uF,\;C_{2}=10\;uF,\;C_{3}=10\;uF,\;C_{4}=10\;uF,\;C_{5}=45\;uF,\;C_{6}=35\;uF,\;L=10\;uH$. A 6 × 6 × 7 periodic circuit is constructed for numerical simulations.

To eliminate the influence of the Dirac point degeneracy in the two-dimensional Lieb lattice when identifying the flat-band response, the system is extended to a three-dimensional Lieb lattice constructed by stacking two-dimensional Lieb lattices along the z direction. The structure of the three-dimensional Lieb lattice is illustrated in Figure 9a [19]. In this lattice, hopping occurs only between different sublattices. The in-plane couplings in the x-y plane are described by the hopping parameters t, $t^{\prime }$, and $J_{1}$, while the interlayer couplings along the z direction are characterized by $J_{1}$ and $J_{2}$. The circuit implementation of the three-dimensional Lieb lattice is shown in Figure 9b. The hopping amplitudes t, $t^{\prime }$, and $J_{1}$ are realized by capacitors *C*_1_ and *C*_3_, respectively. The onsite potentials of sites 1, 2 and 3 are implemented using grounded LC resonant circuits. The interlayer coupling $J_{2}$ is realized using an operational amplifier together with a voltage-controlled current source (VCCS) [34,35], forming a nonreciprocal circuit module, as illustrated in Figure 9c. This configuration allows the implementation of complex hopping coefficients and effectively introduces a Peierls phase in the interlayer coupling [36].

Using the same approach as that employed for calculating the frequency spectrum of the two-dimensional Lieb lattice circuit along the high-symmetry path, Kirchhoff’s law is applied to the unit cell of the three-dimensional Lieb lattice circuit. An infinite three-dimensional periodic lattice is considered, where *q_x_*, *q_y_* and *q_z_* denote the Bloch wave phases propagating along the x, y and z directions, respectively. In the periodic circuit of the three-dimensional Lieb lattice, the voltage vector *V* and the current vector *V* are related through the grounded circuit Laplacian matrix J, which can be written as Equation (22)

(22) $$\left(\begin{array}{c} I_{1}\\ I_{2}\\ I_{3} \end{array}\right)=J\left(\begin{array}{c} V_{1}\\ V_{2}\\ V_{3} \end{array}\right).$$

The admittances of the capacitor and inductor are $y_{C}\;=\;j\omega C$ and ${\;y}_{L}=1/\left(j\omega L\right)$, respectively, where ω  is the angular frequency. The circuit is considered without external sources or sinks, meaning that the total current flowing into each node is zero. Under this condition, the matrix equation can be written as Equation (23)

(23) $$Y\left(\begin{array}{c} V_{1}\\ V_{2}\\ V_{3} \end{array}\right)=\frac{1}{\omega^{2}\;L}\left(\begin{array}{c} V_{1}\\ V_{2}\\ V_{3} \end{array}\right).$$

The admittance matrix *Y* contains the coupling parameters between different nodes as well as the Bloch phase factors arising from the periodic boundary conditions. The imaginary terms in the matrix originate from the nonreciprocal interlayer coupling introduced by the circuit module described above, which effectively produces a phase factor in the interlayer hopping. The admittance matrix *Y* is expressed as Equation (24)

(24) $$J=\left(\begin{array}{ccc} 2C_{1}+2C_{2}+3C_{3}+C_{4}\; & -(C_{1}+C_{2}e^{-iq_{x}}+C_{3}e^{iq_{y}}+iC_{7}e^{-iq_{z}})\; & -(C_{1}+C_{2}e^{-iq_{y}}+2C_{3}\;cos\left(q_{z}\right))\;\\ {-(C}_{1}+C_{2}e^{iq_{x}}+C_{3}e^{-iq_{y}}-iC_{7}e^{iq_{z}}\;) & C_{1}+C_{2}+C_{3}+C_{5}\; & 0\;\\ -(C_{1}+C_{2}e^{iq_{y}}+2C_{3}\;cos\left(q_{z}\right)\;)\; & 0\; & C_{1}+C_{2}+2C_{3}+C_{6} \end{array}\right),$$

**Figure 10:**
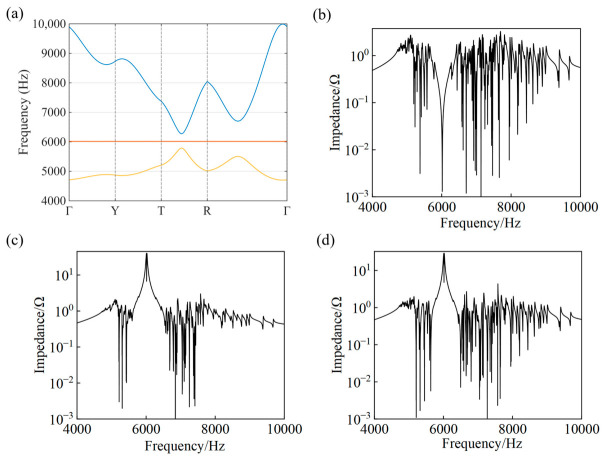
Theoretical frequency spectrum and node impedance responses of the three-dimensional Lieb lattice circuit. (**a**) Eigenfrequency band structure calculated from the grounded circuit Laplacian matrix of a single unit cell derived from the periodic circuit shown in Figure 9b. The band structure is obtained along the high-symmetry path Γ(0,0,0) → X(π,0,0) → T(π,π,0) → R(π,π,π) → Γ(0,0,0) in the Brillouin zone. The circuit parameters are $C_{1}=5\;uF,\;\;C_{2}=10\;uF,{\;C}_{3}=10\;uF,\;C_{4}=10\;uF,{\;C}_{5}=45\;uF,C_{6}=35\;uF,L=10\;uH$. (**b**–**d**) Node-to-ground impedance spectra calculated by applying an AC excitation signal to nodes 1, 2, and 3 of the same circuit unit cell, respectively, using the two-point impedance calculation method.

The circuit parameters are chosen as ${\;C}_{1}=5\;uF,\;C_{2}=10\;uF,\;C_{3}=10\;uF,\;C_{4}=10\;uF,\;C_{5}=45\;uF,\;C_{6}=35\;uF,\;C_{7}=10\;uF,\;L=10\;uH.$ By calculating the eigenvalues of the matrix Y, the corresponding eigenfrequencies ω can be obtained, and the circuit frequencies are determined by f = ω/ 2π. The resulting frequency spectrum along the high-symmetry path Γ(0,0,0) → X(π,0,0) → T(π,π,0) → R(π,π,π) → Γ(0,0,0) in the three-dimensional Brillouin zone is shown in Figure 10a. It can be observed that three bands appear in the spectrum, where the middle band corresponds to a flat band with a frequency of 6015 Hz. In contrast to the two-dimensional case, where the flat band touches the dispersive bands at the Dirac point, the three-dimensional band structure does not exhibit such degeneracy. To investigate the circuit response, the node impedance spectra are calculated at nodes 1, 2, and 3. The impedance spectrum calculated at node 1 is shown in Figure 10b. The impedance–frequency curve exhibits a gap in the flat-band frequency. This behavior can be understood from the perspective of the driving-point impedance; since the flat-band eigenmode has negligible amplitude on node 1, the external excitation couples weakly to this mode, leading to a suppressed impedance response. In contrast, in the impedance spectra calculated at nodes 2 and 3, shown in Figure 10c,d, a pronounced and relatively high peak emerges in the vicinity of the flat-band frequency.

**Figure 11:**
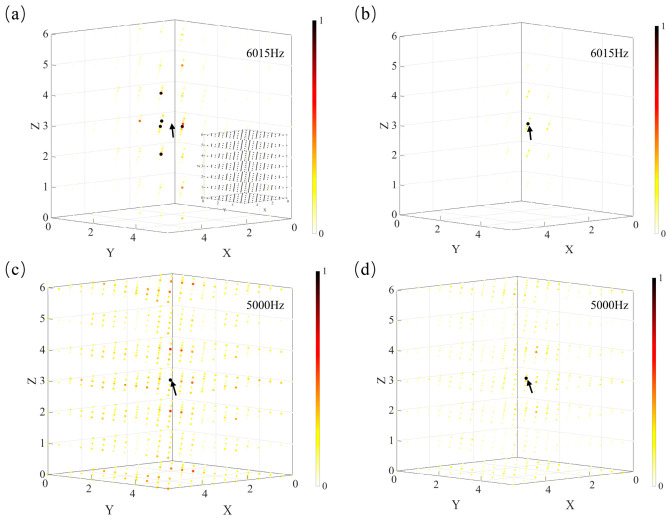
Spatial distributions of normalized node voltage amplitudes in the 6 × 6 × 7 unit-cell periodic three-dimensional Lieb lattice circuit under fixed-frequency excitation. A sinusoidal AC voltage source is applied to excite the circuit, and the steady-state normalized voltage amplitude |*V*| at each node is calculated. One unit cell is selected for illustration. The size of each sphere representing a node is scaled according to the relative magnitude of the node voltage amplitude, with larger spheres indicating higher voltage amplitudes. The lattice coordinates of the two nodes are node 1 (2.5, 2.5, 3) and node 2 (2, 2.5, 3). The position of the excitation point is indicated by the arrow. (**a**) The voltage distribution obtained when node 1 is excited at the flat-band frequency; the bottom-right panel shows the distribution of the circuit nodes. (**b**) Voltage distribution obtained when node 2 is excited at the flat-band frequency. (**c**,**d**) show the voltage distributions obtained when nodes 1 and 2 are excited at 5000 Hz, respectively.

To further investigate the spatial characteristics of the circuit modes, the voltage intensity distributions in the three-dimensional Lieb lattice circuit are calculated under excitation by a fixed-frequency AC source. Here, the coordinates denote the lattice indices of the nodes in the circuit array. In this section, nodes 1 and 2 are selected as the excitation sites. At the flat-band frequency, the voltage distributions obtained under excitation at node 1 and node 2 are shown in Figure 11a,b, respectively. It can be seen that, when the excitation is applied to node 1, almost no appreciable voltage appears at node 1 itself, whereas weak localization can still be observed at the surrounding node 2 and node 3 sites. By contrast, when the excitation is applied to node 2, a much stronger localized feature is clearly observed at node 2. These results further corroborate the impedance-response analysis presented above, indicating that the compact localized states associated with the flat band emerge only on nodes 2 and 3. In addition, when node 1 and node 2 are excited at the off-flat-band frequency of 5000 Hz, the corresponding voltage distributions, shown in Figure 11c,d, exhibit pronounced spreading throughout the circuit and no longer display localized characteristics. Moreover, the calculated IPR values show that, at the flat-band frequency of 6015 Hz, the IPR obtained for excitation at node 1 is 0.05, whereas that obtained for excitation at node 2 is 0.33, indicating a more strongly localized voltage response when the excitation is applied to node 2. In contrast, at the off-flat-band frequency of 5000 Hz, the IPR obtained for excitation at node 1 is 0.004, while that obtained for excitation at node 2 is 0.003, suggesting that the voltage distributions are more extended away from the flat-band resonance.

**Figure 12:**
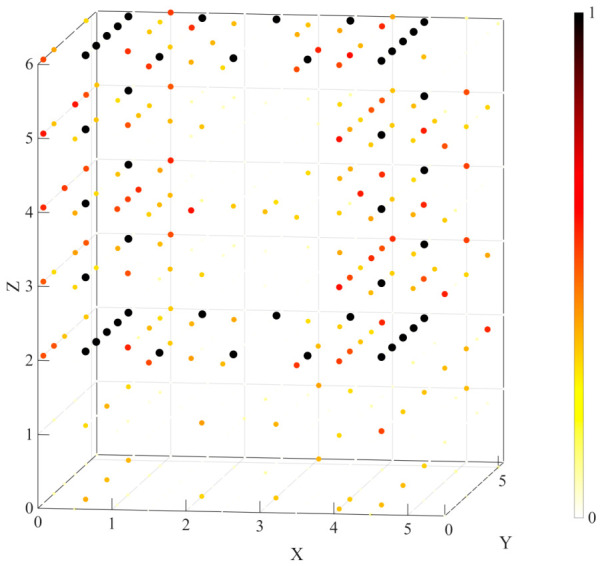
The voltage distribution obtained under excitation in the three-dimensional lattice exhibits a cubic frame-like pattern, where darker colors and larger spheres indicate higher node voltage amplitudes.

In the two-dimensional Lieb lattice, the compact localization associated with flat-band states enables strongly confined voltage distributions when excitations are applied to sublattice 2 and sublattice 3 nodes, which further allows the construction of specific planar patterns such as the letters N, B, and U. Building on this principle, the same strategy is extended to the three-dimensional Lieb lattice circuit. As shown in Figure 12, by employing a similar excitation scheme, a cubic frame-like voltage pattern is realized, with its eight vertices located at (4.5,1,2), (4.5,5,2), (0.5,5,2), (0.5,1,2), (4.5,1,6), (4.5,5,6), (0.5,5,6), and (0.5,1,6). The voltage is predominantly localized at the excited nodes and their nearest neighbors, while the response in other regions is significantly suppressed, demonstrating that the localization mechanism of CLS remains robust across different dimensions. Compared with the two-dimensional case, the voltage distribution in the three-dimensional system is no longer confined to a single plane but extends along the spatial direction, enabling the construction of genuinely three-dimensional voltage patterns. This result indicates that the three-dimensional Lieb lattice not only preserves the localization characteristics of the two-dimensional system but also enables more versatile and controllable spatial mode engineering, offering enhanced flexibility for flat-band-based circuit wavefunction manipulation and information encoding.

## 4. Discussion and Summary

The results show that flat band localization in electrical circuit simulations is shaped not only by the presence of a flat band but also by its degeneracy condition and by momentum sampling in finite systems. The impedance spectra identify the spectral response near the flat band frequency, while the voltage distributions reveal the corresponding spatial localization. Used together, these two quantities give a consistent picture of flat band behavior in the circuit models studied here.

The one-dimensional, two-dimensional, and three-dimensional models therefore illustrate three different situations. In the one-dimensional system, both the impedance spectra and the voltage distributions show clear signatures of localization. In the two-dimensional Lieb lattice, localized responses are still observed near the flat band frequency, but the touching between the flat band and the dispersive bands at the Dirac point leads to hybridization and modifies the observed localization. Under periodic boundary conditions, the discrete sampling of wave vectors also introduces a dependence on whether the number of unit cells is even or odd, because the relevant high symmetry point is included only for certain system sizes. In the three-dimensional Lieb lattice, by contrast, the flat band is separated from the dispersive bands in the spectrum. As a result, hybridization is reduced and the localization behavior becomes clearer, with stronger spatial confinement and clearer sublattice selectivity in the voltage distributions.

It would be interesting to examine how dissipation, component imperfections, and other nonideal circuit effects influence these results. It would also be useful to extend the analysis to other lattice geometries and coupling configurations and to test the predicted localization phenomena in hardware experiments. Such studies may help clarify the broader scope of flat band physics in electrical circuits and support future applications in controllable wave manipulation and circuit design.

In summary, this work constructs one-dimensional and multidimensional flat-band circuit models and systematically investigates their spectral responses and localization properties. By combining node-to-ground impedance spectra with spatial voltage distributions, both the spectral features of flat bands and the structures of their corresponding eigenmodes can be characterized, enabling direct identification of flat-band properties. The results show that the manifestation of flat-band localization depends not only on the existence of the flat band itself but also strongly on band degeneracy and finite-size effects. Specifically, in the two-dimensional Lieb lattice, the flat band touches the dispersive bands at the Dirac point, leading to hybridization with dispersive modes and causing the localization response to deviate from the ideal sublattice-selective behavior. Meanwhile, under periodic boundary conditions, the discrete sampling of wave vectors further introduces parity-dependent responses associated with whether the number of unit cells is even or odd. In contrast, in the three-dimensional Lieb lattice, the absence of degeneracy points separates the flat band from the dispersive bands in the spectrum, allowing its intrinsic localization properties to be manifested more clearly, with stronger spatial confinement and more pronounced sublattice selectivity. These results demonstrate that band degeneracy and system dimensionality play crucial roles in shaping flat-band responses in circuit systems, and further show that electrical circuits provide a highly controllable and flexible platform for investigating geometry-induced localization phenomena. In addition, the pattern-generation results provide an intuitive demonstration of the controllability of flat-band localized modes, showing that localized voltage responses can be selectively excited and spatially arranged through circuit design. In this sense, they support the physical picture of spatial confinement and sublattice selectivity, while also suggesting a possible route toward programable mode manipulation in future circuit systems.

## Data Availability

The original contributions presented in this study are included in the article/Supplementary Material. Further inquiries can be directed to the corresponding author.

## Supplementary Materials

The following supporting information can be downloaded at: https://www.mdpi.com/article/10.3390/ma19101981/s1, Figure S1: High-symmetry points in the frequency spectrum of the two-dimensional Lieb lattice; Figure S2: (a) Impedance measured at node 1 for the single-unit-cell circuit. (b) Impedance measured at node 1 for the 2 × 2 unit-cell circuit. The red region highlights the spectrum in the frequency range from 1091 Hz to 1104 Hz.
