# Supplementary material for: Flat-Band Localization in Electrical Circuits from One to Three Dimensions
Source: Materials (Basel). 2026 May 11;19(10):1981. doi: 10.3390/ma19101981 (PMC13209072; doi:10.3390/ma19101981)
Supplement: Supplementary file 1 [file materials-19-01981-s001.zip › materials-4285945-supplementary.pdf]

Supplementary Material S1

To quantitatively discuss the influence of the band-touching point in the singular flat-band case, a single-unit-cell circuit and a 2×2 unit-cell circuit are selected as representative examples, and their node-impedance spectra are analyzed. As shown in Figure 2, the impedance spectrum measured at node 1 in the single-unit-cell circuit exhibits only two impedance peaks, with no pronounced peak appearing at the flat-band frequency. In contrast, in the 2×2 unit-cell circuit, five peaks are observed in the impedance spectrum, including a distinct peak at the flat-band frequency. To understand this phenomenon, it should be noted that, under periodic boundary conditions, the allowed k values in the circuit are discretely quantized and satisfy the following relation:

$$k_x = \frac{2\pi m}{N_x a_x}, k_y = \frac{2\pi n}{N_y a_y}, \tag{S1}$$

$$m = 0,1,\dots,N_x - 1,\ n = 0,1,\dots,N_y - 1, \tag{S2}$$

where N_x and N_y denote the numbers of unit cells along the (x) and (y) directions, respectively. According to the above relation, the high-symmetry point X(π,π) shown in Figure 1 is included in the allowed discrete (k)-point set only when both N_x and N_y are even. Based on this observation, the single-unit-cell and 2×2 unit-cell circuits can be further analyzed. For the single-unit-cell circuit, since N_x=N_y=1, only one (k) point, namely Γ(0,0), is allowed, and therefore only two peaks are observed in the impedance spectrum measured at node 1. For the 2×2 unit-cell circuit, since N_x=N_y=2, four discrete (k) points are allowed. Taking degeneracies into account, these points can be associated with three high-symmetry points, namely Γ(0,0), X(π,0), and M(π,π).

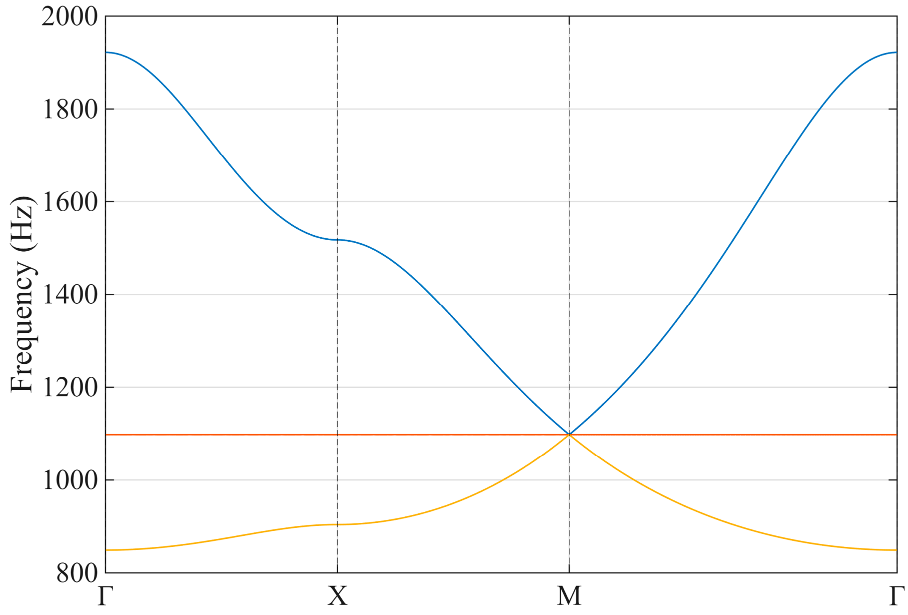

Figure S1. High-symmetry points in the frequency spectrum of the two-dimensional Lieb lattice.

By comparing the peak frequencies measured in Figure 2 with the theoretically calculated eigenfrequencies shown in Figure 1, complete agreement is observed, thereby verifying the validity of the above analysis. It can therefore be further concluded that the flat-band state has no amplitude distribution on node 1. In even-unit-cell systems, the peak appearing at the flat-band frequency for node 1 does not originate from the flat band itself

but rather from the inclusion of the Dirac point in the discrete k-point set. This peak corresponds to the response arising from the projection of the Dirac-point state onto node 1.

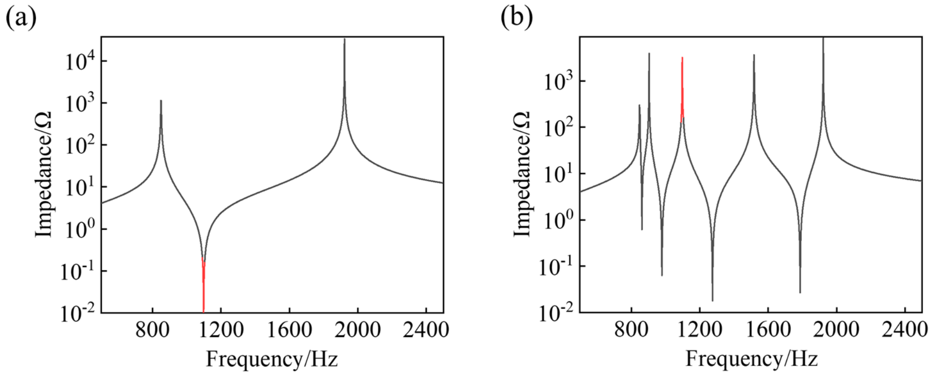

Figure S2. (a) Impedance measured at node 1 for the single-unit-cell circuit. (b) Impedance measured at node 1 for the 2×2 unit-cell circuit. The red region highlights the spectrum in the frequency range from 1091 Hz to 1104 Hz.
